# Fetal Heart Rate Analysis in Pregnancies Complicated by Gestational Diabetes Mellitus: A Prospective Multicentre Observational Study

**DOI:** 10.1111/1471-0528.18010

**Published:** 2024-11-25

**Authors:** Sian Chivers, Caroline Ovadia, Tharni Vasavan, Maristella Lucchini, Barrie Hayes‐Gill, Nicolò Pini, William Paul Fifer, Catherine Williamson

**Affiliations:** ^1^ Department of Women's and Children's Health King's College London London UK; ^2^ Department of Fetal and Paediatric Cardiology Evelina London children's Hospital London UK; ^3^ Department of Obstetric Medicine Guy's and St Thomas' NHS Foundation Trust London UK; ^4^ Department of Psychiatry Columbia University Irving Medical Center New York USA; ^5^ Division of Developmental Neuroscience New York State Psychiatric Institute New York USA; ^6^ Faculty of Engineering University of Nottingham Nottingham UK; ^7^ Institute of Reproductive and Developmental Biology, Imperial College London London UK

**Keywords:** cardiac time intervals, fetal ECG, gestational diabetes, heart rate, heart rate variability, non‐invasive fetal electrocardiography

## Abstract

**Objective:**

Establish whether pregnancies complicated by gestational diabetes mellitus (GDM) are associated with a fetal cardiac phenotype that predisposes to arrhythmia; utilising measurements derived from non‐invasive abdominal fetal ECG.

**Design:**

Prospective observational study.

**Setting:**

Three tertiary obstetric units, United Kingdom.

**Population:**

Women aged ≥ 16 years with either GDM or uncomplicated pregnancy (control) who were > 20 weeks gestational age.

**Methods:**

The MonicaAN24 non‐invasive abdominal fetal ECG monitor was fitted for overnight recording.

**Main Outcome Measure:**

Calculation of the fetal heart rate (FHR) and fetal heart rate variability (HRV) time domain metrics standard deviation of normal‐to‐normal intervals (SDNN), root mean square of successive differences (RMSSD) and the PR, QRS, QT intervals was performed. Groups were compared using linear regression models (stratified by sleep state) and adjusted for fetal sex and ethnicity.

**Results:**

Ninety‐six participants were included. For HRV in sleep state 1F, SDNN was higher for GDM than control participants 12.56 (10.45–16.62)ms versus 8.58 (5.83–9.73)ms [*p* = 0.01] [median (IQR)]. There were no differences in SDNN in sleep state 2F. No differences were identified in RMSSD in either sleep states or in the cardiac time intervals. We observed a negative correlation between HRV and body mass index/HbA1c and a positive correlation between FHR and body mass index/HbA1c in sleep states 1F/2F.

**Conclusions:**

Alterations of HRV and FHR rate may be associated with a diagnosis of GDM, likely secondary to altered autonomic function in utero.

## 1 Introduction

Diabetes mellitus is the most common metabolic disease of pregnancy and is either pre‐existing (type‐1‐diabetes (T1DM) and type‐2‐diabetes (T2DM)) or develops during pregnancy (gestational diabetes mellitus (GDM)). Worldwide, in 2021, it was estimated that 21.1 million (16.7%) of live births had some form of hyperglycaemia in pregnancy. Of these, 80.3% were estimated to have GDM, with the highest comparative prevalence in Southeast Asia when adjusted for maternal age [1]. Adverse perinatal outcomes are associated with diabetes in pregnancy including macrosomia, higher incidence of shoulder dystocia and birth trauma, preterm delivery, congenital heart disease (in pre‐existing diabetes) and stillbirth [2, 3, 4, 5, 6].

Diabetes (T1DM, T2DM, GDM) in pregnancy has known fetal cardiac consequences. Structurally, there is an increased risk of congenital heart disease (CHD) in fetuses of women with pre‐existing diabetes [7]. Additionally, fetuses are at risk of cardiac hypertrophy with associated ventricular dysfunction [8, 9, 10, 11]. Fetal heart rate (FHR) and heart rate variability (HRV) are under autonomic control which may be affected by adverse maternal glucose environment [12]. Prior studies using fetal magnetocardiography (fMCG) demonstrated alterations in FHR and HRV in the fetuses of women with GDM and in those who are overweight/obese signifying autonomic dysfunction [13, 14]. Alterations in fetal HRV have also been shown to have associations with other disease states such as neonatal acidosis following delivery [15]. Atrial arrhythmia has also been reported to have a higher incidence in fetuses of women with diabetes without CHD where there is diastolic dysfunction, independent of the level of ventricular hypertrophy [16].

The main study objective was to establish whether pregnancies complicated by GDM were associated with a fetal cardiac phenotype that was different to that seen in control pregnancies utilising fECG measurements of the FHR, HRV metrics [root mean square of successive differences (RMSSD) and standard deviation of normal‐normal intervals (SDNN)] and the fetal cardiac time intervals (CTIs) [PR, QRS and QT intervals]. We additionally assessed the correlation of these parameters with maternal glucose control measured using glycosylated haemoglobin (HbA1c) and body mass index (BMI) and examined if any periods of abnormal FHR (< 100 bpm/> 200 bpm) were recorded.

## 2 Methods

We prospectively recruited and performed fECG on participants > 20 weeks gestational age (GA) (33.4 (29.3–36.0) weeks [median (IQR)], *n* = 96) with either GDM or an uncomplicated pregnancy (control) who gave written informed consent. Participants were recruited from Guy's and St Thomas' Hospital NHS Foundation Trust, London; Nottingham University Hospital, Nottingham and Queen Charlotte's and Chelsea Hospital, London. Participants were recruited from general antenatal clinics (uncomplicated pregnancies) and pregnancy in diabetes clinics. Ethical approval was obtained: REC:15/WM/0017, REC: 08/H0707/21 and REC:16/LO/0839. Data were collected between 19 March 2015 and 30 September 2020.

The fECG was recorded using the Monica AN24 monitor (Monica Healthcare Limited., Nottingham, UK) for 3.5–15.2 h and worn on one occasion. The Monica AN24 is a handheld device approximately the size of a smartphone, used for ambulatory fECG monitoring. It was fitted by placing five Ambu VLC‐00‐S electrodes on the gravid abdomen. One electrode was placed in the midline, within a range of 5 cm below the uterine fundus, one 5 cm above the symphysis pubis, one on each of the right and left abdominal walls and one reference electrode towards the participants back. The skin was prepared by gentle exfoliation of the surface cells using 3 M Skinprep 2236 at the desired location of electrode placement, prior to placement [17]. The participants had the monitor fitted and tested in the hospital and took the monitor home for the analysis to be performed. They were instructed to switch the monitor on for recording when retiring for the night and remove the monitor when they awoke in the morning. The equipment was collected at the next clinical visit to hospital.

Inclusion criteria were singleton pregnancies with a confirmed diagnosis of GDM and healthy uncomplicated singleton pregnancies. GDM was defined as a blood glucose level of ≥ 5.6 mmol/L fasting or ≥ 7.8 mmol/L at 2 h using the 75 g 2‐h oral glucose tolerance test (OGTT) with testing performed between 24 and 28 weeks gestational age [18]. Exclusion criteria were multifetal pregnancies, participants in active labour and fetuses with known CHD or significant congenital abnormality.

Information was collected on participant demographics (age, ethnicity, gestation at recruitment, medications, family history, past medical and obstetric history), glucose control (fasting blood glucose and HbA1c within three months of recruitment) and neonatal outcome (GA at birth, type of delivery, requirement for admission to the neonatal unit).

The study protocol, use and acceptability of the Monica AN24 for fECG monitoring overnight was determined with pilot studies on healthy pregnant volunteers, and feedback from these and participants from a previous study used to inform the protocol development for this study [19].

### 2.1 Data Analysis

#### 2.1.1 Fetal ECG Data Processing

The fECG recordings were processed using the Monica DK v1.9 software (Monica Healthcare Limited, Nottingham UK). Firstly, the complete FHR signal was extracted and reviewed manually for segments of acquisition where there was suggestion of low maternal movement utilising the inbuilt accelerometer data alongside assessment of acceleration patterns in the maternal heart rate. Following this, a 2‐h period of the recording was chosen where there was an uninterrupted FHR signal to perform HRV analysis and measurement of the CTIs within this time window. The first and last hours of the recording were not included in the analysis to reduce selection of time periods where there was maternal wakefulness.

#### 2.1.2 Fetal Behavioural State Coding

The FHR expressed in beats per minute (bpm) was plotted as function of time and coded using a fetal behavioural state coding program in Matlab (version R2017a) (MathWorks Inc. USA) previously used to assess the HRV patterns of fetuses enrolled in the safe passage study where 1655 fetuses were investigated [20]. Behavioural state was classified as 1F (quiet sleep), 2F (active sleep), 3F (quiet awake), 4F (active awake) or ‘indeterminable’. Given that the recordings were performed overnight in the participants own home, we did not have access to ultrasound data on fetal body and eye movements and so behavioural state was coded on FHR patterns alone. A minimum of 3 min duration is required for any state to be reliably defined [21]. Expert training was provided to TV and SC by the researchers who developed the algorithm to validate competence in the assessment of fetal behavioural state and use of the algorithm.

#### 2.1.3 Heart Rate Variability Analysis

The healthy heart does not beat uniformly like a metronome. Instead, there are fluctuations between heartbeats (HRV), enabling the body to cope with uncertain and challenging environments. There are several methods for the assessment of HRV for which we have utilised the time‐domain methods in this study known as SDNN and RMSSD. SDNN comprises measurement of the standard deviation of inter‐beat intervals between successive normal R‐R intervals, with abnormal beats such as ectopic beats removed from analysis. The RMSSD is calculated through assessment of each successive time difference between heartbeats. Each value is squared, and the mean value is calculated. Thereafter the square root of this value is obtained giving the RMSSD. Both SDNN and RMSSD measurements are reported in milliseconds (ms) [22].

In this study fetal R‐R intervals were extracted from the raw fECG using MonicaDK v1.9. as a .csv file. The custom Matlab script was used to assess the quality of the recording in 30 s epochs. Epochs were included in the analysis if the quality was rated as > 70% in the two‐hour window. Data quality was defined as the number of accepted R waves divided by the total number of R waves within an epoch. Fetal R‐R intervals were acceptable if > 300 ms and < 667 ms and the absolute difference between consecutive R‐R intervals was < 10% [23]. The mean R‐R interval from each 30 s epoch was derived utilising the Matlab script and from these values SDNN and RMSSD were computed. Behavioural state coding was applied following this to categorise the data.

#### 2.1.4 Cardiac Time Interval Analysis

A signal‐averaged fECG complex was generated via MonicaDK v1.9 from the fetal signal (following extraction from the maternal ECG (mECG) by MonicaDK v1.9) using a heart rate range of 120–160 bpm. Signals outside of this range were excluded because of the association between low and high FHR's and arrhythmia which could produce an abnormal complex where the CTIs may not be measured accurately. Where there were multiple areas of usable fECG acquisition, several sections with good acquisition as defined above were assessed, and one signal‐averaged complex with the clearest points of interest visually was chosen for analysis. The fiducial points (P, Q, R, S, T) were marked by two independent researchers (SC/TV) and the intervals of interest (PR, QRS and QT) were derived from these markings. Inter‐observer reliability between manual measurements of the investigators was calculated with an intraclass coefficient of 0.80 for the PR interval, 0.81 for the QRS interval and 0.72 for the QT interval.

### 2.2 Statistical Analysis

Statistical analysis and graphs were performed using Stata17 (StataCorp. 2021.College Station, TX: StataCorp LLC). Data were aggregated and presented as median (IQR) and n/N(%) for continuous and categorical data respectively, *p* < 0.05 were considered statistically significant. Demographic and outcome data were compared using the Mann–Whitney test. Fetal ECG data were compared using linear regression. Both non‐adjusted and adjusted values for fetal sex and ethnicity were calculated and *p*‐values, regression coefficients and 95% confidence interval of the coefficient are presented. Pearson's correlation was used to compare two continuous variables.

## 3 Results

### 3.1 Clinical and Demographic Details of Participants

One hundred and six participants were enrolled in the study from four UK obstetric units. Of these, 96 were included in the analysis due to ten recordings being unsuccessful and providing no interpretable data. Median (IQR) age of participants was 34 (30–37) years. Demographic data (age, pregnancy number, recruitment gestation, body mass index (BMI) and blood pressure (BP)) showed no significant difference between groups, except ethnicity where there were a higher proportion of white participants amongst the control group (89.7%) when compared with the GDM group (44.7%). Maternal medical/obstetric history in 17 individuals comprised of multiple conditions including: anxiety/depression in five, structural gynaecological disease in five, previous GDM in two, asthma in two and systemic lupus erythrobates, epilepsy, ulcerative colitis, migraine, previous premature birth and ectopic pregnancy in one each.

The majority of patients with GDM had relatively mild disease [24], with normal fasting blood glucose < 5.1 mmol/L (18/32, [56%]) and HbA1c below 36 mmol/mol (19/35, [54%]). Of those with GDM, 19/38 (50%) were managed using diet alone, 9/38 (24%) were taking metformin only, 8/38 (21%) were managed with metformin and insulin and 2/38 (5%) were managed with insulin only. Clinical and demographic details are shown in Table 1.

**TABLE 1:** Demographic and outcome data of participants within the study.

	GDM (*n* = 38)	Control (*n* = 58)	*p*
Age [median (IQR), *n* =]	33 (29–38), *n* = 37	35 (32–36), *n* = 26	0.18
Pregnancy number [median (IQR), *n* =]	2 (1–3), *n* = 36	2 (1–2), *n* = 23	0.05
Recruitment gestation [median (IQR), *n* =]	34.0 (30.0–36.1), *n* = 38	33.9 (30.3–36.9), *n* = 56	0.74
BMI [median (IQR), *n* =]	24.2 (22.6–28.2), *n* = 36	23.8 (22.0–26.6), *n* = 43	0.43
Fasting glucose (mmol/L) [median (IQR), *n* =]	4.9 (4.6–5.4), *n* = 32	4.5 (4.2–5.3), *n* = 12	0.32
Fasting glucose < 5.1 (mmol/L) [*n*/*N*, %]	18/32, (56%)	7/12, (58%)	
HbA1c (mmol/mol) [median (IQR), *n* =]	34 (31–38), *n* = 35	34 (32–34), *n* = 14	0.78
HbA1c < 36 (mmol/mol) [*n*/*N*, %]	19/35, (54%)	11/12, (92%)	
BP systolic [median (IQR), *n* =]	110 (101–120), *n* = 36	113 (100–119), *n* = 19	0.63
BP diastolic [median (IQR), *n* =]	67 (60–74), *n* = 36	65 (62–73), *n* = 19	0.88
Ethnicity			**< 0.001**
White [frequency (%)]	17/38 (44.7)	52/56 (92.9)	
Black [frequency (%)]	5/38 (13.2)	3/56 (5.4)	
Asian [frequency (%)]	7/38 (18.4)	1/56 (1.8)	
Chinese [frequency (%)]	5/38 (13.2)	0/56 (0)	
Mixed [frequency (%)]	2/38 (5.3)	0/56 (0)	
Other [frequency (%)]	2/38 (5.3)	0/56 (0)	
Gestational age at birth [median(IQR), *n* =]	38 (38–39), *n* = 38	39.6 (39.0–40.6), *n* = 52	**0.001**
Induced [frequency (%)]	18/35 (51.4)	5/40 (12.5)	**< 0.001**
Male sex [frequency (%)]	20/35 (57.1)	29/53 (54.7)	0.83
Birthweight (g) [median(IQR), *n* =]	3160 (2940–3570), *n* = 35	3365 (3068–3730), *n* = 52	0.20
Birthweight centile [median(IQR), *n* =]	43.0 (22.0–73.9), *n* = 35	49.5 (18.2–71.0), *n* = 52	
Apgar 1 min [median(IQR), *n* =]	9 (9–9), *n* = 38	9 (9–9), *n* = 58	0.90
Apgar 5 min [median(IQR), *n* =]	10 (9–10), *n* = 38	10 (9–10), *n* = 58	0.15
Meconium present [frequency (%)]	4/35 (11.4)	5/31 (16.1)	0.59
Neonatal unit admission [frequency (%)]	2/35 (5.7)	0/35 (0)	0.23
Mode of delivery			0.56
Unassisted vaginal birth [frequency (%)]	16/35 (45.7)	28/52 (53.8)	
Assisted vaginal birth [frequency (%)]	5/35 (14.3)	4/52 (7.7)	
Elective Caesarean birth [frequency (%)]	9/35 (25.7)	15/52 (28.8)	
Unplanned Caesarean birth [frequency (%)]	5/35 (14.3)	5/52 (9.6)	

Abbreviations: BMI = body mass index, BP = blood pressure, GDM = Gestational diabetes mellitus, HBA1c = glycosylated haemoglobin, IQR = interquartile range. Bold value indicates statistically significant.

Of the 96 participants included in the study, 42/96 (44%) had data from sleep state 1F, 81/96 (84%) had data from sleep state 2F and 89/96 (93%) had cardiac time interval data of sufficient quality for analysis.

### 3.2 Fetal Heart Rate

FHR data are shown in Table 2. In sleep state 1F FHR was 127 (123–133) bpm [*n* = 20] in pregnancies where there was GDM and 130 (125–134) bpm [*n* = 21] in controls. In sleep state 2F, FHR was 135 (132–139) bpm [*n* = 34] in pregnancies where there was GDM and 135 (130–140) bpm [*n* = 46] in controls. Difference was not detected in FHR between pregnancies complicated by GDM and controls in sleep states 1F or 2F both unadjusted and adjusted for fetal sex and maternal ethnicity. No periods of significant tachycardia or bradycardia signifying possible arrhythmia were seen on the study recordings.

**TABLE 2:** Fetal heart rate, cardiac time intervals and heart rate variability in participants with type 1 diabetes compared with control pregnancies.

	GDM (*n* = 38)	Control (*n* = 58)	Unadjusted			Adjusted		
			*p*	Regression Coefficient	95% CI	*p*	Regression Coefficient	95% CI
Heart rate (bpm)								
FHR (1F) Median (IQR)	127 (123–133) *n* = 20	130 (125–134) *n* = 21	0.54 *n* = 41	−1.70	−7.20 to 3.80	0.88 *n* = 39	−0.50	−7.29 to 6.30
FHR (2F) Median (IQR)	135 (132–139) *n* = 34	135 (130–140) *n* = 46	0.54 *n* = 80	0.99	−2.16 to 4.13	0.56 *n* = 75	1.16	−2.77 to 5.08
Heart rate variability (ms)								
SDNN 1F Median (IQR)	12.56 (10.45–16.62) *n* = 20	8.58 (5.83–9.73) *n* = 21	**0.01** ***n* = 41**	**4.04**	**0.94–7.14**	**0.01** ***n* = 39**	**5.42**	**1.55–9.30**
RMSSD 1F Median (IQR)	7.58 (7.20–9.21) *n* = 20	8.71 (7.62–9.93) *n* = 21	0.38 *n* = 41	−0.45	−1.49 to 0.58	0.53 *n* = 39	−0.41	−1.71 to 0.89
SDNN 2F Median (IQR)	22.57 (19.50–26.19) *n* = 34	18.79 (15.41–25.65) *n* = 46	0.31 *n* = 80	1.74	−1.63 to 5.11	0.30 *n* = 75	2.18	−1.98 to 6.33
RMSSD 2F Median (IQR)	8.86 (7.91–9.48) *n* = 34	8.93 (7.60–10.12) *n* = 46	0.81 *n* = 80	−0.10	−0.90 to 0.71	0.47 *n* = 75	−0.37	−1.38 to 0.64
Cardiac time intervals (ms)								
PR interval Median (IQR)	107 (93–112) *n* = 35	108 (102–114) *n* = 45	0.10 *n* = 80	−4.50	−9.94 to 0.94	0.19 *n* = 74	−4.57	−11.48 to 2.34
QRS duration Median (IQR)	56 (51–57) *n* = 35	54 (51–59) *n* = 45	0.83 *n* = 80	−0.28	−2.90 to −2.33	0.86 *n* = 74	0.30	−2.97 to 3.56
QT interval Median (IQR)	262 (250–276) *n* = 35	276 (258–289) *n* = 45	**0.02** ***n* = 80**	−11.05	−20.39 to −1.72	0.26 *n* = 74	−6.65	−18.32 to 5.02

*Note:* Fetal heart rate [bpm], cardiac time intervals [ms] and heart rate variability metrics (ms) in participants with type 1 diabetes compared with control pregnancies. p‐values, regression coefficients and 95% confidence intervals of the coefficient adjusted for fetal sex and ethnicity.

Abbreviations: 1F = fetal sleep state 1F, 2F = fetal sleep state 2F, bpm = beats per minute, FHR = fetal heart rate, GDM = gestational diabetes mellitus, IQR = interquartile range, ms = milliseconds, RMSSD = root mean square of successive differences, SDNN = standard deviation of normal‐to‐normal intervals. Bold value indicates statistically significant.

### 3.3 Maternal GDM and Fetal Heart Rate Variability Analysis

**FIGURE 1:**
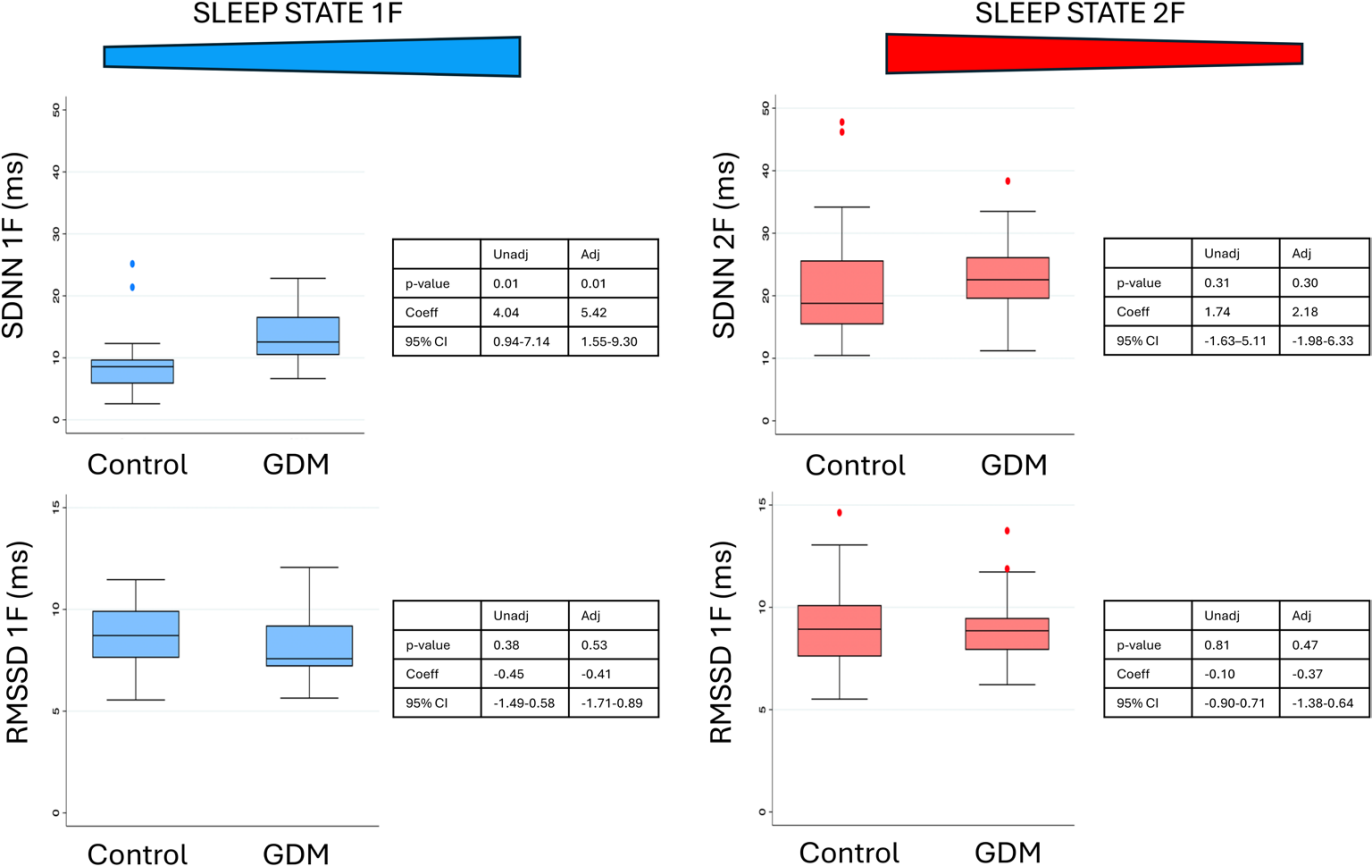
Time domain heart rate variability measurements standard deviation of normal‐to‐normal intervals (SDNN) [ms] and root mean square of successive differences (RMSSD) [ms] in both quiet (sleep state 1F) and active (sleep state 2F) sleep. Data presented for both uncomplicated pregnancies and those complicated by gestational diabetes mellitus. 1F = fetal sleep state 1F, 2F = fetal sleep state 2F, Adj = adjusted value, bpm = beats per minute, FHR = fetal heart rate, GDM = gestational diabetes mellitus, IQR = interquartile range, ms = milliseconds, MSSD = root mean square of successive differences, SDNN = standard deviation of normal‐to‐normal intervals, Unadj = unadjusted value.

Fetal HRV comparability are shown in Figure 1 and data in Table 2. In sleep state 1F, SDNN (ms) was 12.56 (10.45–16.62) [*n* = 20] in the GDM group and 8.58 (5.83–9.73) [*n* = 21] in the control group, and RMSSD (ms) was 7.58 (7.20–9.21) [*n* = 20] in the GDM group and 8.71 (7.62–9.93) [*n* = 21] in the control group. In sleep state 2F the SDNN (ms) was 22.57 (19.50–26.19) [*n* = 34] in the GDM group and 18.79 (15.41–25.65) [*n* = 46] in the control group, and RMSSD (ms) was 8.86 (7.91–9.48) [*n* = 34] in the GDM group and 8.93 (7.60–10.12) [*n* = 46] in the control group. In sleep state 1F the SDNN was higher (both unadjusted and adjusted for fetal sex and ethnicity) in the GDM group and there was no certainty of a difference in sleep state 2F or in RMSSD in both sleep states 1F and 2F as the confidence intervals contained both clinically important increases and decreases.

### 3.4 Maternal GDM and Fetal Cardiac Time Interval Analysis

**FIGURE 2:**
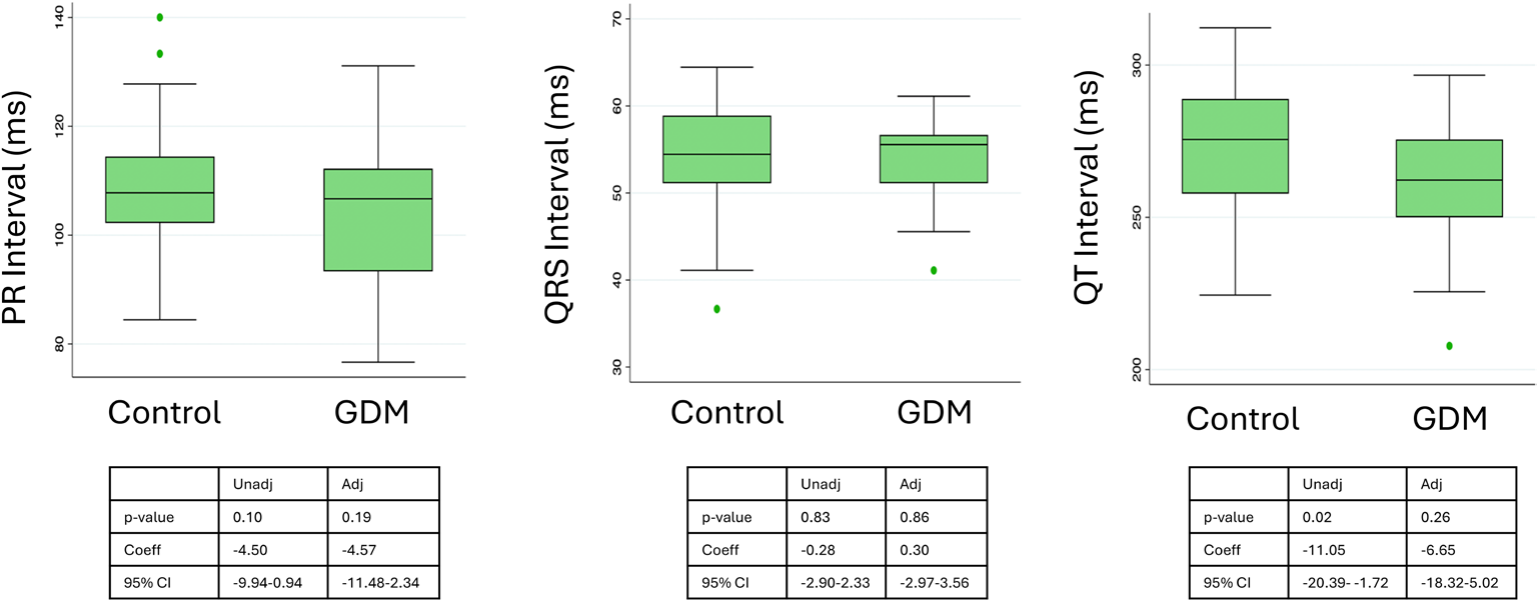
Cardiac time intervals measured by the non‐invasive abdominal fECG in uncomplicated pregnancies compared to those complicated by gestational diabetes mellitus. Panel A—PR interval, Panel B—QRS interval, Panel C—QT interval. Adj = adjusted value, GDM = gestational diabetes mellitus, ms = milliseconds, Unadj = unadjusted value.

Fetal cardiac time interval comparisons are shown in Figure 2 and data in Table 2. PR interval (ms) was 107 (93–112) [*n* = 35] in the GDM group and 108 (102–114) [*n* = 45] in the control group. QRS duration (ms) was 56 (51–57) [*n* = 35] in the GDM group and 54 (51–59) [*n* = 45] in the control group. QT interval (ms) was 262 (250–276) [*n* = 35] in the GDM group and 276 (258–289) [*n* = 45] in the control group. Unadjusted, there was no difference between groups for the PR and QRS intervals, however, a difference was found in the QT interval between groups, with shorter QT interval seen in GDM pregnancies. Adjusted for fetal sex and ethnicity no differences were seen.

### 3.5 Correlation With Body Mass Index and HbA1c

**FIGURE 3:**
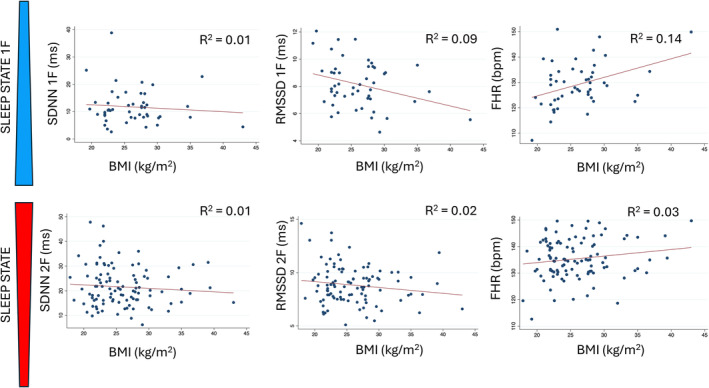
Correlation between body mass index (BMI) and fetal heart rate (FHR) [bpm] / heart rate variability parameters (SDNN and RMSSD) [ms]. Graphs are divided into sleep state 1F (quiet sleep) shown in the top row and 2F (active sleep) shown in the bottom row. 1F = fetal sleep state 1F, 2F = fetal sleep state 2F, bpm = beats per minute, FHR = fetal heart rate, GDM = gestational diabetes mellitus, IQR = interquartile range, ms = milliseconds, RMSSD = root mean square of successive differences, SDNN = standard deviation of normal‐to‐normal intervals.

**FIGURE 4:**
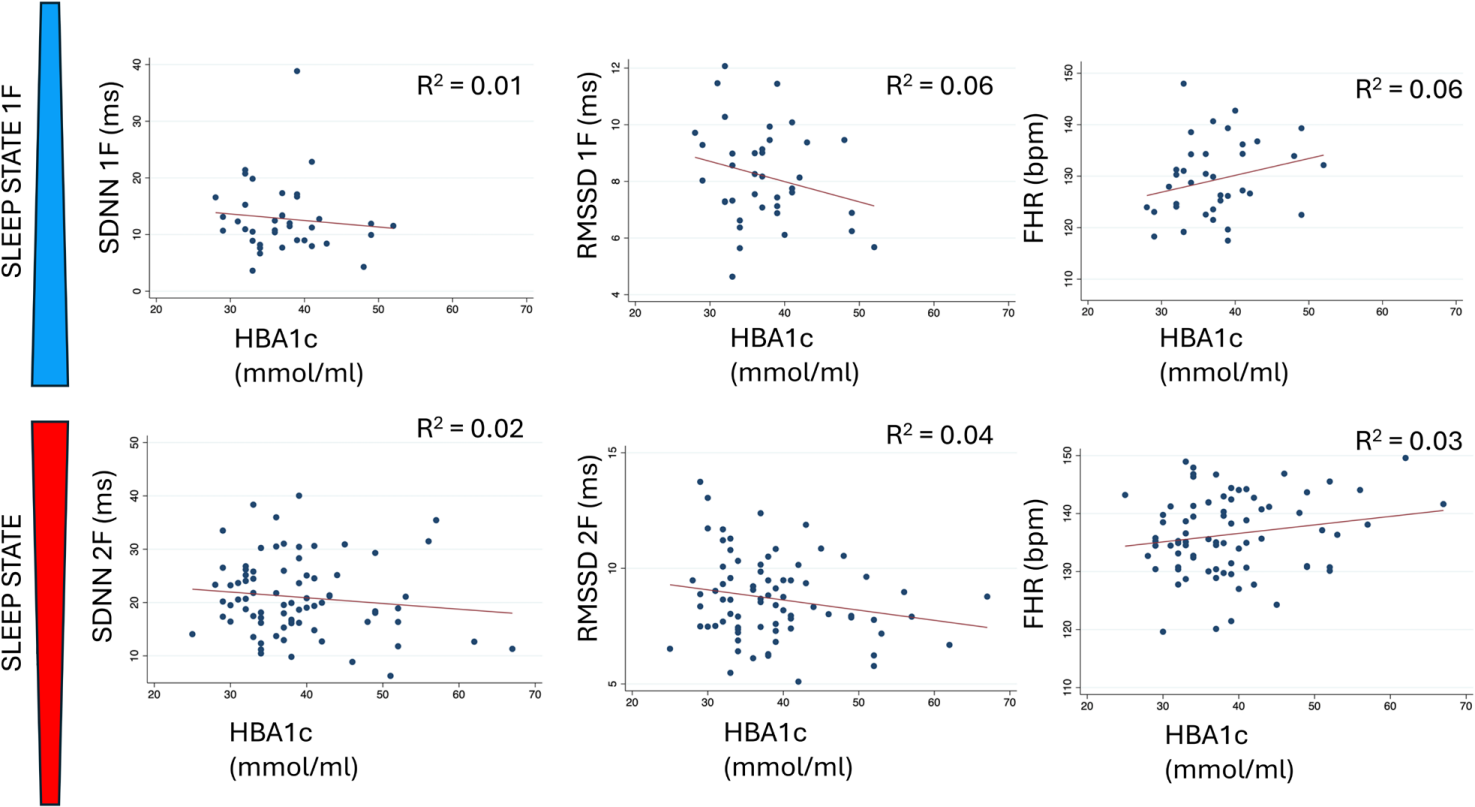
Correlation between HbA1c and fetal heart rate (FHR) [bpm] / heart rate variability parameters (SDNN and RMSSD) [ms]. Graphs are divided into sleep state 1F (quiet sleep) shown in the top row and 2F (active sleep) shown in the bottom row. 1F = fetal sleep state 1F, 2F = fetal sleep state 2F, bpm = beats per minute, FHR = fetal heart rate, GDM = gestational diabetes mellitus, IQR = interquartile range, ms = milliseconds, RMSSD = root mean square of successive differences, SDNN = standard deviation of normal‐to‐normal intervals.

Measures of HRV (SDNN and RMSSD) and HbA1c showed a negative correlation with BMI, most notably RMSSD in sleep state 1F. FHR showed a positive correlation with BMI and HbA1c (Figures 3 and 4).

### 3.6 Neonatal Outcomes

Birthweight, birthweight centile and neonatal outcomes (Apgar, presence of meconium and neonatal unit admission) were similar between groups. There were higher rates of induction in the group with GDM (51.4%) compared with controls (12.5%). There was also a difference in birth gestation between groups but both groups had a median gestation that was term (> 37 weeks). Data are presented in Table 1.

## 4 Discussion

### 4.1 Main Findings

In this study on the effect of GDM on the fetal heart rhythm, overall, we did not have certainty on the difference in fetal HRV between groups. Differences were not seen in the FHR and CTIs. However, there were associations evident between maternal BMI and HbA1c and the FHR patterns, with a negative correlation between HRV and BMI/HbA1c and a positive correlation between FHR and BMI/HbA1c. These findings add to the evolving literature on the effect of diabetes on the developing heart and autonomic nervous system.

### 4.2 Interpretation

In fetal life, altered HRV responses have been shown in several studies utilising fMCG. Fehlert et al. [13] assessed HRV using fMCG in pregnant women with GDM and normal glucose tolerance during an oral glucose tolerance test (OGTT). After the oral glucose load, in control pregnancies the HRV parameters almost continuously rose, whereas in the GDM group, they initially rose and then decreased to below baseline at 120 min. A further study by Zöllkau et al. [25] investigated 19 pregnancies complicated by GDM and compared these with 167 controls. They found an increase in vagal modulation of heart rate patterns during active sleep (sleep state 2F) characterised by increases in RMSSD and high frequency power measures. Our results did not reveal the same findings. A possible explanation for this discrepancy is that the majority of patients with GDM in our cohort had mild disease, and maternal BMI did not differ between the GDM and control groups. Therefore, as the fECG was performed at 34 weeks GA (median), there may have been sufficient time after the diagnosis of GDM (which is typically made at 24–28 weeks GA) for lifestyle and medication factors to have taken effect to improve the metabolic dysfunction that may predispose to FHR and HRV abnormalities in GDM.

When our results of HRV and FHR were correlated against HbA1c and BMI they showed a negative correlation in HRV, most notably in RMSSD in sleep state 1F (quiet sleep). Additionally, a positive correlation was seen for FHR. These findings are consistent with the findings from Fehlert et al. [13], with lower HRV at 120 min after oral glucose load, and where overall mean FHR was higher in fetuses of mothers with GDM compared to those with normal glucose tolerance [17]. Furthermore, it shows consistency with Zöllkau et al. [25] who reported a positive correlation between maternal glucose levels and sympathetic activity (measured by SDNN). The effect of maternal BMI was additionally investigated by Mat Husin et al. [14] using fMCG in 184 pregnancies. They also showed that there was increased FHR in mothers with pre pregnancy overweight or obesity and reduced HRV in mothers with high maternal weight gain. These findings further support the possible explanation that severity of metabolic disturbance influences the changes on the cardiac and autonomic nervous systems.

A difference was seen in SDNN in sleep state 1F but not in sleep state 2F, or in RMSSD. The change in SDNN in sleep state 1F demonstrated greater HRV in fetuses from pregnancies complicated by GDM compared to controls. Previous studies have hypothesised that alterations in HRV occur secondary to changes in autonomic function with a tendency towards sympathetic predominance. Russell et al. [26] compared 38 neonates born to mothers with T1DM and 24 controls using a 40‐min postnatal ECG recording. They found a difference in HRV with increased overall power in the frequency domain analysis proportionally in the low frequency spectrum reflecting sympathetic overactivity in the cohort exposed to T1DM in pregnancy. The findings from this study showed increased SDNN in the T1DM group, but no changes in RMSSD. The participants with T1DM had higher mean HbA1c levels (> 6.1%/43 mmol/mol) compared with our cohort. These findings may reflect that a greater phenotypic change is seen in presence of more pronounced metabolic difference between groups. This study included frequency domain analyses. Addition of frequency and non‐linear metrics could provide researchers and clinicians with a richer picture of the fetal HRV patterns in different disease states.

In this study the measurements SDNN and RMSSD were used to assess HRV. These time domain measurements are frequently used but form part of a wider spectrum of HRV analysis. Whist the time domain metrics can be quantified into high and low, more advanced assessment of physiological state can be gained by combining these measures with the other components of HRV. HRV metrics are grouped into linear (comprising time and frequency domain) and non‐linear models. Frequency domain metrics estimate the distribution of absolute or relative power into frequency bands using autoregressive techniques or fast Fourier transformations. Using these techniques, one can assess whether high or low frequency signals predominate within a sample [22].

Non‐linear models are the third component of HRV. These enable quantification of the unpredictability of a time‐series resulting from the complexity of the mechanisms that regulate HRV. Examples of non‐linear analyses include, correlation dimension, entropy, detrended fluctuation analysis and fractal analysis. Whilst these have not been investigated as part of this study, they have been widely explored in a research environment including in patients with diabetes and cardiovascular disease [22, 27]. One study assessing cardiovascular health in adults with diabetes investigated time, frequency and non‐linear HRV and showed that most non‐linear parameters were higher in normal subjects compared to those with diabetes. Higher non‐linear HRV may however not signify good health, with other studies in people post myocardial infarction and the elderly with higher non‐linear metrics in the groups with worse outcomes [28].

None of the fetuses investigated in this study were diagnosed with arrhythmia antenatally, and no evidence of abnormal FHR that could signify arrhythmia was identified using the monitor. There were no clinically important differences between groups in the cardiac time intervals when adjusted for baseline characteristics; alterations in these intervals can signify development of arrhythmia and conduction abnormalities as seen in long‐QT syndrome and heart block secondary to circulating maternal Ro/La antibodies. Other investigators have suggested that tachyarrhythmia including atrial flutter and ectopic atrial tachycardia has a higher incidence in fetuses and neonates of women with diabetes, including one single centre study where 26% of 31 fetuses or neonates presenting with atrial flutter or ectopic atrial tachycardia had a history of maternal diabetes in pregnancy (75% of whom had GDM). The authors hypothesised that these findings were secondary to diastolic dysfunction causing atrial stretch, as seen in the adult population [16]. The non‐invasive abdominal fECG monitor could provide further opportunity to study this potential association and additional utility as a screening tool in high‐risk pregnancies, for example where there is known cardiac hypertrophy.

The fECG's performed in this study were undertaken as overnight recordings because from a technical perspective this is the time where there is least maternal movement artefact and highest signal quality can be obtained. Whilst we were able to obtain high quality signal, consideration should be made as to how the results may change during the daytime secondary to fluctuations in maternal heart rate and glucose load from meals. Fehlert et al. [13] investigated the response to oral glucose load and found lower HRV at 120 min in those with GDM highlighting that fetal exposure may be an important consideration when extrapolating data to daytime.

## 5 Strengths and Limitations

This prospective study adds information to the literature on the effect of GDM on the developing heart rhythm and raises the important question that severity of disease in pregnancies complicated by diabetes could be an important factor influencing fetal cardiac development. A limitation of this study is that control participants were from a less ethnically diverse group of women than the GDM group. Further studies from other international centres including low‐income countries would provide more in‐depth information on the impact of GDM on the fetal heart in different environments and populations. Additionally, a focussed study investigating the fetus where there is increased risk of stillbirth in the context of GDM, such as those who are large for gestational age [29] may provide valuable insight into the aetiology of adverse outcomes in this group.

## 6 Conclusions

Our findings revealed no overall differences in HRV and FHR in pregnancies complicated by GDM in a population where there was no baseline difference in HbA1c and BMI between groups. There was a negative correlation between HRV and a positive correlation between FHR and BMI/HbA1c suggesting that alterations in the in utero metabolic environment led to changes in cardiac and autonomic metrics, as seen in other studies. This study emphasises the importance of glycaemic control in women with diabetes in pregnancy and how, if controlled, this can enable reassurance by clinicians.

Further studies could lead to developments in this area by focussing on the relationship between severity of metabolic derangement in GDM and changes in the fECG and could also investigate pregnancies complicated by pre‐existing diabetes using the fECG and response to therapy. Given that large amounts of data can be obtained by overnight monitors, investigation of ways to analyse and present the whole dataset in terms of both HRV and visualisation of the ECG trace on a beat‐to‐beat basis would give more informative information on the underlying heart rhythm. Advances in real time monitoring technology where data can be transmitted as it occurs could provide early markers of cardiac activity and timely alerts in pregnancies complicated by diabetes.

## Author Contributions

S.C., C.O. and C.W. designed the study. S.C., T.V. and M.L. collected and analysed the data. S.C., C.O., B.H.G., N.P., W.P.F. and C.W. interpreted the data. S.C. wrote the manuscript and did the figures. All authors reviewed and approved the final manuscript.

## Conflicts of Interest

Professor Williamson is a consultant to GSK and Mirum Pharmaceuticals (payment made to King's College London). Dr. Barrie Hayes‐Gill was the Research Director at Monica Healthcare Ltd. (designer and manufacturer of the AN24) From May 2005 to July 2018. Since this date BHG has not had any formal engagement with Monica Healthcare Ltd. This manuscript was planned after July 2018.

## Data Availability

The data that support the findings of this study are available from the corresponding author upon reasonable request.
